# From Chronic Anemia to Total Gastrectomy: A Case Report on a Challenging Form of Ménétrier’s Disease

**DOI:** 10.7759/cureus.88724

**Published:** 2025-07-25

**Authors:** Malak Faiz, Soraya Gioftsiou, Fatine ElGraoui, Zineb Malki, Mohamed Mohammadi

**Affiliations:** 1 Department of Hepatogastroenterology, Cheikh Zaid University Hospital, Rabat, MAR

**Keywords:** chronic anemia, foveolar hyperplasia, gastric fold hypertrophy, hypertrophic gastropathy, ménétrier’s disease, polypoid gastric lesions, protein-losing gastropathy, total gastrectomy

## Abstract

Ménétrier’s disease (MD) is a rare hypertrophic gastropathy marked by enlarged gastric folds, excessive mucus secretion, and protein-losing enteropathy. We present the case of a 25-year-old man initially hospitalized with severe chronic anemia, which revealed a rare and challenging form of MD. The patient reported persistent epigastric pain, vomiting, and recurrent hematemesis. Biological workup showed profound hypoalbuminemia (20 g/L) and microcytic anemia (Hb 5.8 g/dL, ferritin 3 ng/mL). Abdominal CT and upper endoscopy revealed massive polypoid thickening of the gastric fundus with cerebral-like folds and friable polypoid lesions. Histology confirmed foveolar hyperplasia with glandular atrophy, consistent with MD, in the absence of *Helicobacter pylori* infection.

Despite supportive care, the patient experienced recurrent gastrointestinal bleeding and transfusion-dependent anemia, ultimately requiring total gastrectomy. Surgery led to a significant improvement in symptoms and nutritional status.

The case report emphasizes the diagnostic complexity and challenges of managing MD, particularly in patients with severe presentations. The successful outcome following total gastrectomy underscores the importance of considering surgical options in selected cases.

## Introduction

Ménétrier’s disease (MD) is a rare acquired gastric disorder characterized by hypertrophic gastropathy, excessive mucus secretion, and significant protein loss. First described in 1888 by the French pathologist Pierre Ménétrier, its incidence and mortality rate remain poorly defined. It primarily affects middle-aged men and presents with abdominal pain, nausea, vomiting, anemia, hypochlorhydria, and peripheral edema due to exudative enteropathy [1].

Histologically, MD is characterized by giant hypertrophic rugae, massive foveolar hyperplasia, glandular atrophy, and an altered crypt-to-gland ratio in the gastric mucosa. In children, the disease is generally benign and self-limiting, often associated with cytomegalovirus (CMV) infection. In adults, however, it follows a slow and chronic course. Although its exact etiology remains uncertain, some studies suggest possible links with *Helicobacter pylori* and CMV, as well as the involvement of transforming growth factor in its pathogenesis [1,2].

Therapeutic strategies, including *H. pylori* eradication, corticosteroids, octreotide, and monoclonal antibodies, have shown variable outcomes. Additionally, an association between MD and an increased risk of gastric cancer has been observed, highlighting the need for further research to better understand its pathophysiology and optimize management [3].

## Case presentation

A 25-year-old male patient was hospitalized for severe anemia. He reported a history of a treated peptic ulcer disease two years prior, which required multiple blood transfusions, but had no other medical history. His father had a history of pancreatic cancer. For the past two months, he had been experiencing epigastric pain associated with vomiting and episodes of hematemesis. Biological tests revealed hypoalbuminemia and iron-deficiency anemia (Table 1).

**Table 1:** Biological findings

Test	Result	Normal range
Albumin	20 g/L	35-50 g/L
Hemoglobin	5.8 g/dL	13.8-17.2 g/dL (men)
Ferritin	3 ng/mL	12-300 ng/mL

**Figure 1:**
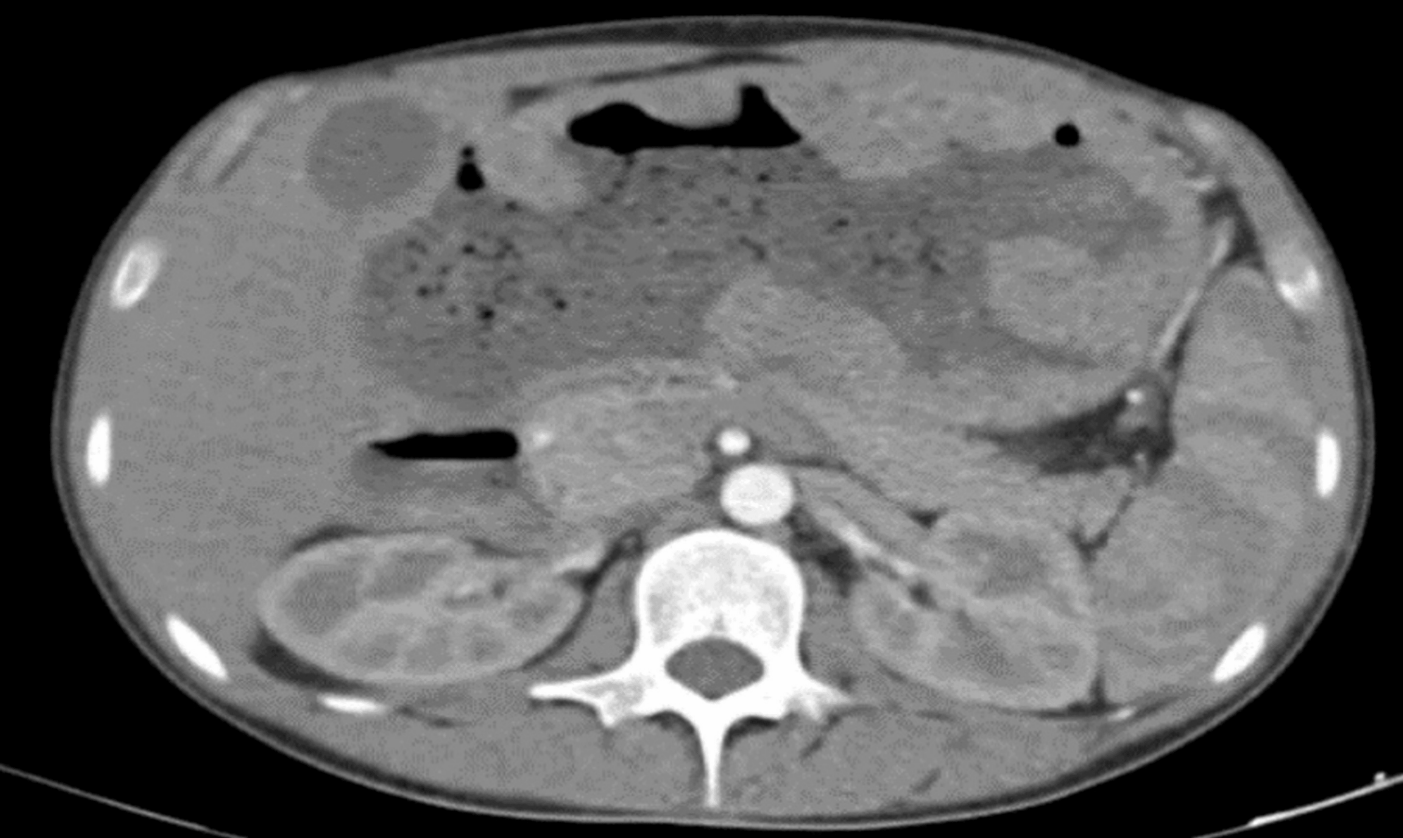
Axial contrast-enhanced CT images showing marked hypertrophy of the gastric folds, predominantly involving the fundus and body of the stomach

**Figure 2:**
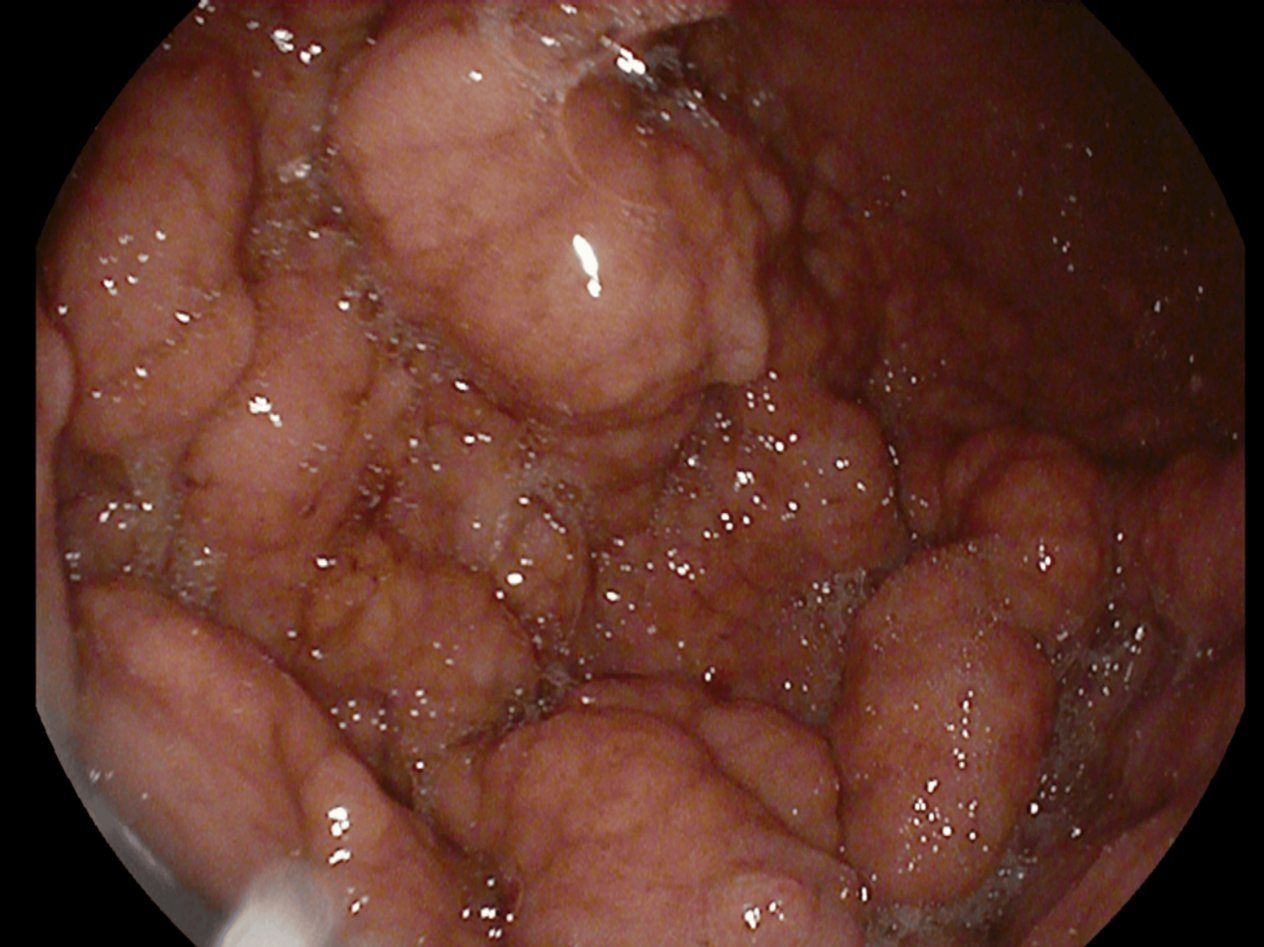
Endoscopic image showing hypertrophic gastritis

**Figure 3:**
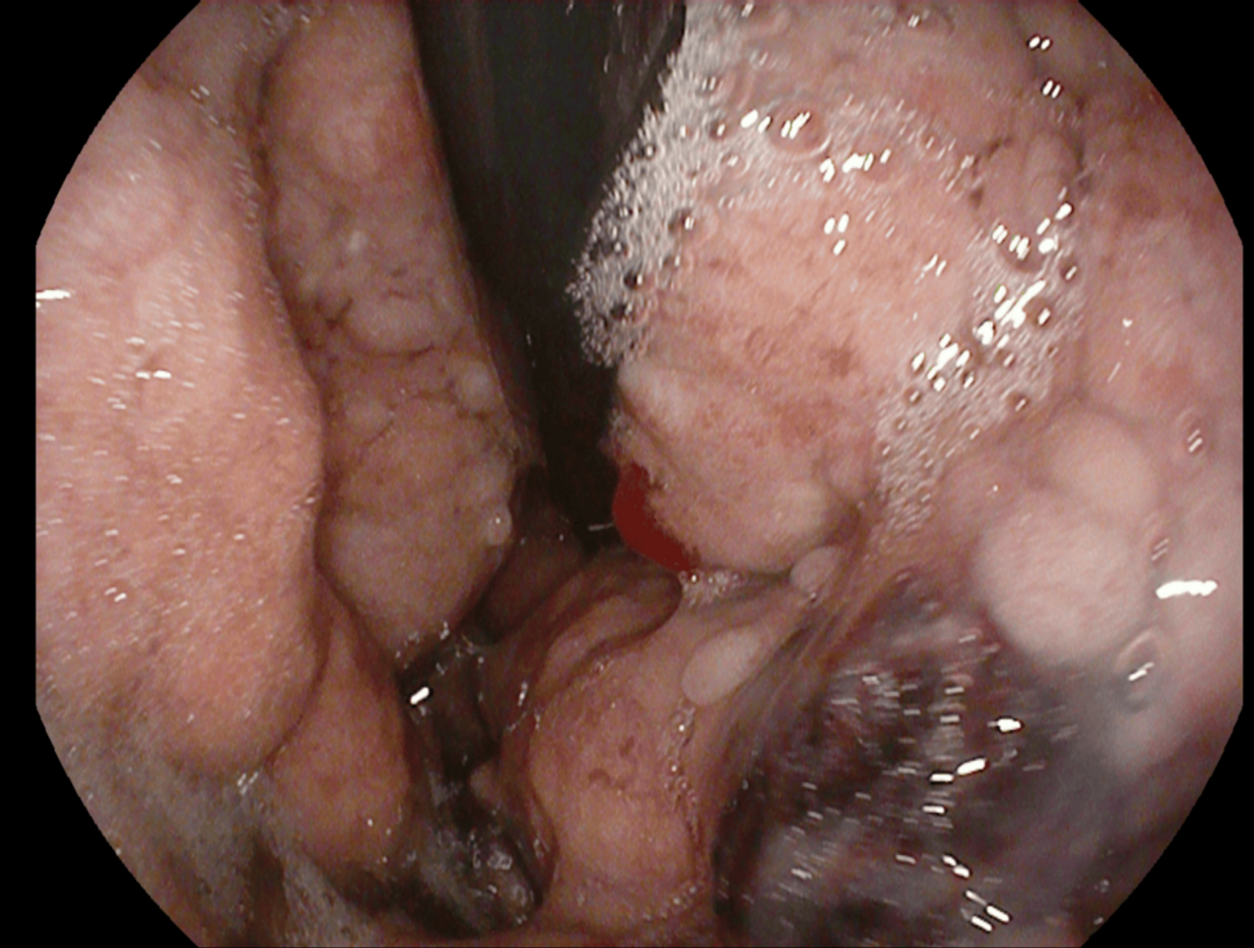
Endoscopic image showing hypertrophic gastritis with signs of bleeding

**Figure 4:**
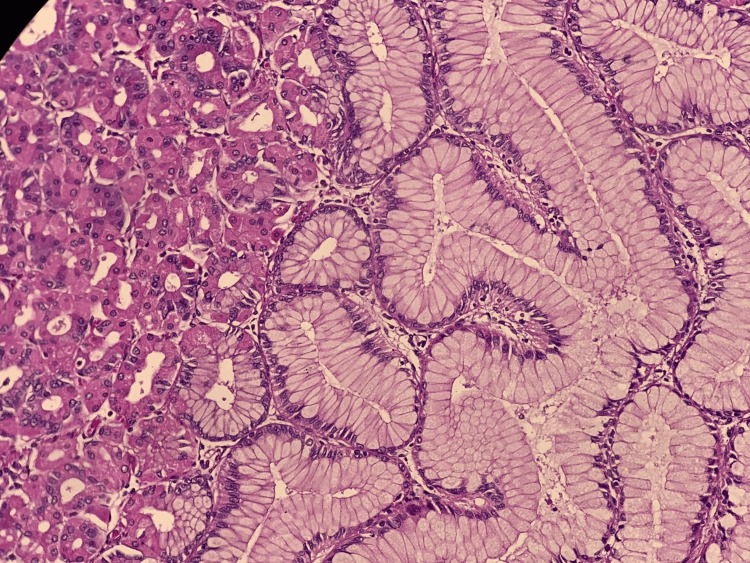
Histological aspect of Menetrier's disease on the gastrectomy specimen

An abdominal CT angiography showed significant polypoid gastric formations occupying the upper two-thirds of the stomach while sparing the pyloric region and perigastric fat, suggesting MD (Figure 1). Upper gastrointestinal (GI) endoscopy revealed cerebral-like hypertrophy of the fundic folds with multiple sessile polypoid lesions that bled upon contact (Figures 2, 3). Histopathological examination demonstrated marked foveolar hyperplasia with glandular atrophy and mononuclear inflammatory infiltration, confirming hypertrophic gastropathy (MD) with no evidence of *H. pylori* infection (Figure 4).

Due to recurrent anemia and active bleeding, a hemostatic gastrectomy was indicated. The patient underwent surgery successfully, resulting in improved quality of life.

## Discussion

MD is an uncommon hypertrophic gastropathy characterized by striking enlargement of gastric folds, diffuse foveolar hyperplasia, and glandular atrophy, leading to marked protein loss and resultant hypoalbuminemia. Clinically, patients often report persistent epigastric pain, frequent vomiting, and upper GI bleeding, sometimes manifesting as melena or hematemesis. These symptoms, combined with generalized edema due to protein-losing gastropathy, significantly impair quality of life. Laboratory findings typically include hypoalbuminemia and iron-deficiency anemia, with hypo- or achlorhydria confirmed on gastric pH studies [4].

The pathogenesis remains incompletely understood. In adults, a notable subset of patients (up to 20% in recent case reviews) are positive for *H. pylori*, and resolution following eradication therapy has been documented, suggesting a potential causative link. However, many cases, like this one, are *H. pylori*-negative, prompting exploration of other mechanisms. Central to disease development is the overexpression of transforming growth factor-alpha (TGF-α) and subsequent activation of the epidermal growth factor receptor (EGFR) in gastric mucosa. Experimental models overexpressing TGF-α replicate MD histological features, including mucosal thickening, mucus hypersecretion, and parietal cell loss [4,5].

Beyond protein loss, MD can be complicated by persistent bleeding due to erosive mucosal damage, severe anemia, and, uncommonly, gastric outlet obstruction or gastroduodenal intussusception. Importantly, a long-term risk for progression to gastric adenocarcinoma exists, with observational studies reporting malignancy in approximately 6%-10% of adult cases over 10 years. Consequently, regular endoscopic surveillance with biopsies is advised in management guidelines [5].

Initial management strategies emphasize supportive care: high-protein diets, proton pump inhibitors to reduce acid secretion, and correction of electrolyte and nutritional deficiencies. For *H. pylori*-positive patients, eradication therapy can induce clinical remission [6,7].

More targeted medical treatments include EGFR inhibitors: cetuximab, for example, has demonstrated the capacity to reduce gastric mucosal thickness, improve serum albumin levels, and alleviate symptoms in small clinical series. Other adjuncts such as octreotide or corticosteroids have shown variable success in symptom relief and reduction of protein loss in limited reports [7].

When conservative therapies fail, particularly in cases of refractory bleeding, severe anemia, or unacceptable symptom burden, surgical intervention is warranted. Total gastrectomy has repeatedly been documented as a highly effective measure in resolving gastropathy, halting protein loss, and restoring nutritional status. Numerous case reports describe dramatic postoperative improvement in both clinical symptoms and laboratory parameters [8].

In this specific case, the combined presence of persistent severe anemia and recurrent upper GI bleeding despite multiple therapeutic attempts justified the choice of total gastrectomy. Subsequent postoperative follow-up demonstrated stabilization of hemoglobin and albumin levels, resolution of GI bleeding, and a marked improvement in functional status, underscoring the merit of personalized management decisions based on disease severity, response to treatment, and risk of complications.

## Conclusions

MD is a rare gastric disorder characterized by gastric fold hypertrophy, foveolar hyperplasia, and hypoalbuminemia. This case illustrates the severity of its complications, particularly GI bleeding and severe anemia, necessitating appropriate management. Despite the absence of *H. pylori* infection, the clinical course rapidly progressed to a surgical indication due to recurrent bleeding. Total gastrectomy improved the patient's quality of life, confirming its role as a last-resort treatment for severe and medically refractory cases. This case highlights the importance of early diagnosis and a multidisciplinary approach to optimize patient outcomes in this rare disease.

## References

[1] Scharschmidt BF (1977). The natural history of hypertrophic gastropathy (Menetrier's disease): report of a case with 16 year follow-up and review of 120 cases from the literature. Am J Med.

[2] Bluth RF, Carpenter HA, Pittelkow MR, Page DL, Coffey RJ (1995). Immunolocalization of transforming growth factor-alpha in normal and diseased human gastric mucosa. Hum Pathol.

[3] Badov D, Lambert JR, Finlay M, Balazs ND (1998). Helicobacter pylori as a pathogenic factor in Ménétrier's disease. Am J Gastroenterol.

[4] Baumeister C, Hüneburg J (2025). Ménétrier disease: a scoping review of case reports over the last 10 years. Bratisl Med J.

[5] Fiske WH, Tanksley J, Nam KT (2009). Efficacy of cetuximab in the treatment of Menetrier's disease. Sci Transl Med.

[6] Parianos C, Aggeli C, Sourla A, Zografos GN (2020). Total gastrectomy for the treatment of Menetrier's disease persistent to medical therapy: a case report. Int J Surg Case Rep.

[7] Wolfsen HC, Carpenter HA, Talley NJ (1993). Menetrier's disease: a form of hypertrophic gastropathy or gastritis?. Gastroenterology.

[8] Toubia N, Schubert ML (2008). Menetrier's disease. Curr Treat Options Gastroenterol.

